# Simple and flexible classification of gene expression microarrays via Swirls and Ripples

**DOI:** 10.1186/1471-2105-11-452

**Published:** 2010-09-08

**Authors:** Stuart G Baker

**Affiliations:** 1 Biometry Research Group, Division of Cancer Prevention, National Cancer Institute, EPN 3131, 6130 Executive Blvd MSC 7354, Bethesda, MD 20892-7354, USA

## Abstract

**Background:**

A simple classification rule with few genes and parameters is desirable when applying a classification rule to new data. One popular simple classification rule, diagonal discriminant analysis, yields linear or curved classification boundaries, called Ripples, that are optimal when gene expression levels are normally distributed with the appropriate variance, but may yield poor classification in other situations.

**Results:**

A simple modification of diagonal discriminant analysis yields smooth highly nonlinear classification boundaries, called Swirls, that sometimes outperforms Ripples. In particular, if the data are normally distributed with different variances in each class, Swirls substantially outperforms Ripples when using a pooled variance to reduce the number of parameters. The proposed classification rule for two classes selects either Swirls or Ripples after parsimoniously selecting the number of genes and distance measures. Applications to five cancer microarray data sets identified predictive genes related to the tissue organization theory of carcinogenesis.

**Conclusion:**

The parsimonious selection of classifiers coupled with the selection of either Swirls or Ripples provides a good basis for formulating a simple, yet flexible, classification rule. Open source software is available for download.

## Background

### Simplicity and flexibility

Simple classification rules with few variables and parameters are preferable to complicated classification rules for the following two reasons [1]. First, classification performance is primarily a function of the first few variables selected, with only slight improvements when additional variables are included. Second, only those variables that strongly predict class in the study data, namely the first few selected, are also likely to moderately or strongly predict class in new data. A popular simple classification rule for analyzing gene expression microarrays is diagonal discriminant analysis, which is discriminant analysis with a diagonal variance-covariance matrix [2]. Diagonal discriminant analysis yields linear or curved classification boundaries, given the name Ripples. Although Ripples are optimal boundaries for normally distributed expression levels in each class with the appropriate variance [3], they can perform poorly with other distributions of gene expression levels [4]. A simple modification of diagonal discriminant analysis with two classes yields a smooth highly nonlinear classification boundary, given the name Swirls. Swirls can outperform Ripples under certain scenarios. The proposed simple, yet flexible, classification rule for two classes selects either Swirls or Ripples after parsimoniously selecting the number of genes and the distance measure.

Classification rules generally have two objectives: prediction and understanding [5], which correspond to Goals 1 and 2, respectively, which are described below.

### Goal 1: Rule discovery and testing

Rule discovery and testing involves splitting the data once into training and test samples, selecting the classification rule in the training sample, and evaluating the performance of this classification rule in the test sample. A univariate measure of performance when the training sample is "fixed", as in this case, is called the conditional error [6]. Two measures of performance that are more informative than the conditional error are the receiver operating characteristic (ROC) curve and the relative utility (RU) curve. The ROC curve plots true versus false positive rates. The RU curve plots the maximum expected utility of prediction as a fraction of the utility of perfect prediction versus the risk threshold, which is the risk corresponding to indifference between harms and benefits [7,8].

### Goal 2. Gene discovery

Gene discovery involves identifying those genes that contribute most to good classification by repeatedly randomly splitting the data into training and test samples, computing a distribution of ROC curves in the test samples to ascertain classification performance, and tabulating the most frequently selected genes in the training sample [9,10].

### Data Sets

Applications of the proposed methodology with both goals involve the following five publicly available data sets for gene expression microarrays:

Colorectal cancer: 2000 genes, 22 normal and 40 tumor specimens [11],

Leukemia 1: 7219 genes, 47 ALL, and 25 AML specimens [12],

Medulloblastoma: 7129 genes, 39 survivor and 21 non-survivor specimens [13],

Prostate cancer: 12,600 genes, 52 tumor and 50 non-tumor specimens [14],

Leukemia 2: 12625 genes, 43 T-ALL specimens and 79 TEL-AML specimens [15].

## Results

### Classification Rule

Let *j *index gene, and *k = *0 and 1 index class. The following quantities specify the classification rule:

(1) $$\begin{array}{l} F=\text{classification components = (}C\text{, }G\text{, }D\text{, }S\text{),}\;\text{where}\\ C=\text{centroid set}=\;\text{\{}c_{jk}\text{,}v_{jk}\text{,}n_{k}\text{\},}\\ \;c_{jk}=\text{centroid}=\;\text{mean expression level for gene }j\text{ in class }k,\\ \;v_{jk}=\text{estimated variance of expression level for gene }j\text{ in class }k,\\ \;n_{k}=\text{number of specimens in class }k,\\ G\;=\;\text{gene set,}\\ D\;=\;\text{distance measure fiom specimen to centroid,}\\ S=\;\text{score formula for combining distance measures}\text{.} \end{array}$$

Let *z*_*hj *_denote the set of expression level of gene *j *in new specimen *h*, and let *Z*_*h *_= {*z*_*hj*_} denote the set of expression levels for specimen *h*. The distance from specimen *h *to the centroid of class *k*, based on gene set *G*, is

(2) $$\text{Distance}(Z_{h},\;k)=\{\begin{array}{cc} \sqrt{\sum_{j\in \text{G}}{\left(z_{hj}-c_{jk}\right)}^{2}/v_{jP},} & \text{if}\;D=1,\\ \sqrt{\sum_{j\in \text{G}}{\left(z_{hj}-c_{jk}\right)}^{2}/v_{jk},} & \text{if}\;D=2, \end{array}$$

where *v*_*jP *_= {(*n*_0 _-1) *v*_*j*0 _+ (*n*_1 _-1) *v*_*j*1_}/(*n*_0 _+ *n*_1 _-2) is the pooled variance over the two classes. Thus *D *is the distance measure with

*D *= 1 = Euclidean distance divided by the pooled variance,

*D *= 2 = Euclidean distance divided by the class-specific variance.

Division by the variance ensures that the distance measure is not inappropriately weighted by genes with high average levels of expression. The score for combining distance measures is

(3) $$\text{Score}(Z_{h},\;F)=\{\begin{array}{ll} \text{Distance}\;{\left(Z_{h},\;0\right)}^{2}\;-\text{Distance}\;{\left(Z_{h},\;1\right)}^{2}, & \text{if}\;S\;=1,\\ \frac{\text{Distance(}Z_{h}\text{,0)}}{\text{Distance(}Z_{h}\text{,0)}+\text{Distance(}Z_{h}\text{,1)}}, & \text{if}\;S\;=2. \end{array}$$

Thus *S *indicates the score formula, either a difference of squared distances or a fraction of the total of the distances. The classification rule for specimen *h*, which is based on the cutpoint *u *of the score, is

(4) $$\text{Rule}(Z_{h},F,u)=\{\begin{array}{ll} \text{assign}\;Z_{h}\;\text{to}\;\text{class}\;1, & \text{if}\;\text{Score}(Z_{h},F)\geq u,\\ \text{assign}\;Z_{h}\;\text{to}\;\text{class}\;0, & \text{otherwise}. \end{array}$$

Diagonal linear and quadratic discriminant analysis correspond to *S *= 1 with *D *= 1 and *D *= 2, respectively [2]. A classification rule with *S *= 2 and Euclidean distance was previously used to analyze microarrays [10] but without a discussion of its implications.

### Swirls and Ripples

**Figure 1:**
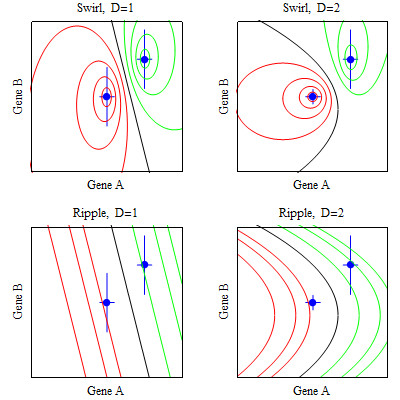
**Illustrative classification boundaries for two genes**. The points are the centroids. Vertical and horizontal lines at the centroid are proportional to the variances. Distance measures are *D *= 1 = pooled variance and *D *= 2 = class-specific variance.

By setting equation (3) equal to various constants and plotting the solution, one can see that the score formula *S *= 2 yields a classification boundary that encircles a centroid and the score formula *S *= 2 yields a boundary of lines and curves (Figure 1). These boundary shapes motivate the following terminology for the score formula,

*S *= 1 = Ripples,

*S *= 2 = Swirls.

**Figure 2:**
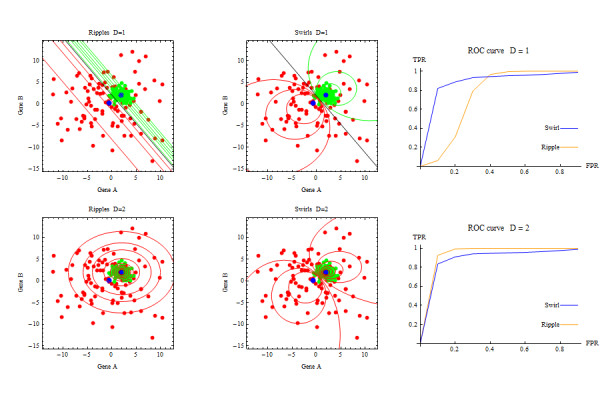
**Swirls and Ripples applied to data generated with *D *= 2**.

If the data in each class are normally distributed, Ripples is the optimal classification boundary if the correct variance (pooled or class-specific) is specified [3]. However if the data in each class are normally distributed with class-specific variances, and one specifies a pooled variance to avoid adding parameters, then Swirls can dramatically outperform Ripples (Figure 2). The proposed classification rule selects either Swirls or Ripples, which increases flexibility without adding parameters.

### Implementation

The proposed classification rule involves randomly splitting 70% of the data into a training sample and 30% into a test sample. Classification performance in the training sample is measured via the area under the ROC curve, denoted AUC, computed assuming a normal distribution of scores in each class. Details are provided in the Methods Section.

For each score formula and distance measure *D*, the classification rule selects a gene set *G *by first identifying the 50 highest ranking genes in terms of AUC of the score and, after starting with the highest ranking gene, successively including genes from these 50 highest ranking genes until there is little improvement classification performance. A greedy algorithm, which is sometimes called forward stepwise selection, successively adds the gene that most improves classification performance given the previously selected genes in the classification rule. For the greedy algorithm, the classification rule adds a gene only if the increase in AUC of the score is at least 0.02. A wrapper algorithm selects features by "wrapping around" (invoking) the full method of selecting a classification rule that uses both training and test samples, when these samples are nested within the training sample [16]. The wrapper algorithm randomly splits half the training sample data into training-training and training-test samples, which is repeated five times. On each random split, a greedy algorithm within the wrapper algorithm formulates a classification rule based on centroid set in the training-training sample and the gene expression levels in the training-test sample, successively adding a gene to the classification rule only if the increase in AUC of the score is at least 0.01. The wrapper algorithm selects the best performing classification rule among classification rules obtained in the five random splits. (Although the wrapper algorithm makes use of a greedy algorithm, reference to a greedy algorithm, unless otherwise noted, means the greedy algorithm not embedded in the wrapper algorithm). After the classification rule computes the gene set *G *as described above, for each score formula *S *the classification rule selects *D *= 1 if the increase in AUC with *D *= 2 is less than 0.02, and selects *D *= 2 otherwise. Finally the classification rule selects the score formula, *S *= 1 =Ripples or *S *= 2 = Swirls, that has the highest AUC.

Computations for Goal 1 are based on a distribution of ROC curves in the test sample computed from 20 bootstrap iterations. The RU curve is derived from the concave envelope of the mean ROC curve over the bootstrap iterations. Computations for Goal 2 are based on 100 random splits into training and test samples.

### Simulation

**Figure 3:**
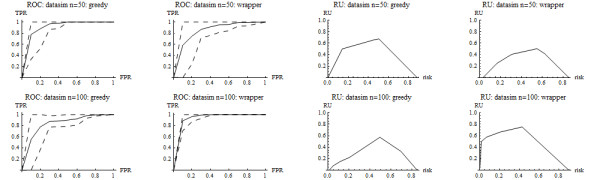
**ROC and RU curves for simulation**.

Simulations are useful for investigating some aspects of classification rules, but one should not overly rely on their results because little is known about the true distributions of gene expression levels [17]. Here a simulation was used to investigate the ability to identify informative genes in a simple setting. The simulation involved 2002 genes with independent normal distributions including (*i*) 2000 non-informative genes with mean 0 and standard deviation 5 in each class and (*ii*) 2 informative genes, used for Figure 2, each with mean 0 and standard deviation 5 in class 0 and mean 2 and standard deviation 1 in class 1. Sample sizes were 50 and 100 per class. For Goal 1, the classification rule under the wrapper algorithm included both informative genes and performed well for both sample sizes In contrast, the classification rule under the greedy algorithm included only one informative gene and performed poorly with a sample size of 50 per class and performed well with a sample size of 100 per class (Tables 1 and 2 and Figure 3). For Goal 2, the two informative genes were selected much more frequently than the non-informative genes (Table 3), as anticipated.

**Table 1:** Classification rules selected from simulated data using a greedy algorithm.

			Gene set = *G*		Centroid set = *C*			
			
Sample size	*S*	*D*	*j*	Description	***c***_***j0***_	***c***_***j1***_	***v***_***j0***_	***v***_***j1***_
*n*_*k *_= 50	Swirl	1	2002	informative	-0.8	2.0	4.4	4.4

*n*_*k *_= 100	Swirl	2	2002	informative	-0.1	2.1	5.5	0.9

			2001	informative	0.1	1.9	5.1	0.9

			162	non-informative	0.5	-0.7	5.0	5.5

			1118	non-informative	-1.4	0.9	5.2	5.0

**Table 2:** Classification rules selected from simulated data using a wrapper.

			Gene set = *G*		Centroid set = *C*			
			
Sample size	*S*	*D*	*j*	Description	***c***_***j0***_	***c***_***j1***_	***v***_***j0***_	***v***_***j1***_
*n*_*k *_= 50	Swirl	2	2002	Informative	-2.2	1.7	6.5	1.0

			1771	non-informative	-0.9	2.5	3.9	6.7

*n*_*k *_= 100	Swirl	2	2002	Informative	1.1	2.3	5.7	0.8

			7	non-informative	0.4	0.4	5.0	6.5

			2001	Informative	-0.3	2.0	5.3	0.9

			323	non-informative	-0.3	1.0	5.1	6.0

**Table 3:** Most frequently selected genes in simulated data.

		Gene		
			
Sample size	Feature selection	*j*	Description	Percentage of splits
*n*_*k *_= 50	Greedy	2002	informative	48%

		2001	informative	20%

		1565	non-informative	4%

	Wrapper	2002	informative	48%

		2001	informative	14%

		707	non-informative	3%

*n*_*k *_= 100	Greedy	2001	informative	38%

		2002	informative	27%

		996	non-informative	4%

	Wrapper	2002	informative	26%

		2001	informative	26%

		1941	non-informative	1%

### Data analysis

**Figure 4:**
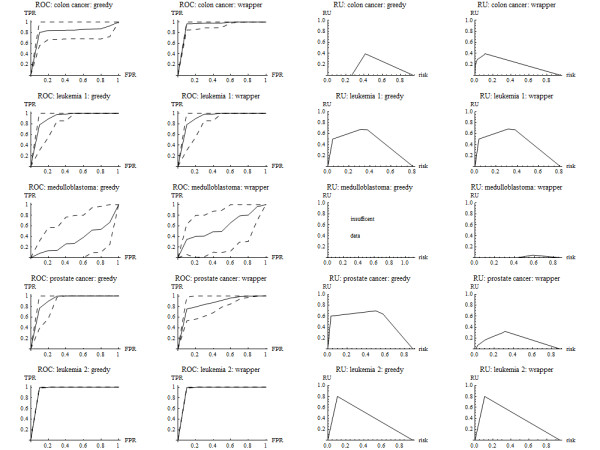
**ROC and RU curves for data sets**.

For Goal 1, the classification rules under both greedy and wrapper algorithms performed well in all data sets except for medulloblastoma (Tables 4 and 5 and Figure 4). For Goal 2, there was good classification in test samples obtained by random splits in all data sets except for medulloblastoma (not shown). The most frequently occurring genes among random splits of the training sample associated with good classification were desmin, zyxin, hepsin, and HLA class II. See Table 6.

**Table 4:** Classification rules selected in data sets using greedy algorithms.

			Gene set = *G*		Centroid set = *C*		
			
Data set	*S*	*D*	*j*	Description	***c***_***j0***_	***c***_***j1***_	***v***_***jP***_
Colon cancer	Swirl	1	493	myosin heavy chain	716	278	338

Leukemia 1	Swirl	1	3532	glutathione S-transferase	81	1456	449

Medulloblastoma	Swirl	1	6230	myosin heavy polypeptide 7	0	-53	234

Prostate cancer	Swirl	1	6185	serine protease hepsin	48	184	70

Leukemia 2	Swirl	1	8828	HLA class II alpha	1504	40640	7151

**Table 5:** Classification rules selected in data sets using a wrapper.

			Gene set = *G*		Centroid set = *C*		
			
Data set	*S*	*D*	*j*	Description	***c***_***j0***_	***c***_***j1***_	***v***_***jP***_
Colon cancer	Swirl	1	1772	myosin heavy chain	44	125	61

			249	desmin	1958	467	793

			1582	p cadherin	53	174	83

			1423	myosin reg light chain 2	763	196	213

			745	ORF, xq terminal portion	188	226	179

Leukemia 1	Swirl	1	3532	glutathione S-transferase	37	1460	569

Medulloblastoma	Swirl	1	977	zinc finger protein HZfq	38	-102	87

Prostate cancer	Swirl	1	8850	cDNA DKFZp564A072	24	215	110

Leukemia 2	Swirl	1	8828	HLA class II alpha	785	41345	7280

**Table 6:** Genes most frequently selected in data sets.

		Gene		
			
Data set	Feature selection	*j*	Description	Fraction of splits
Colon cancer	Greedy	249	**desmin**	0.17

		493	myosin heavy chain	0.15

		1772	collagen alpha 2	0.12

	Wrapper	249	**desmin**	0.13

		1772	collagen alpha 2	0.08

		1582	p cadherin	0.06

Leukemia 1	Greedy	4847	**zxyin**	0.46

		6855	TCF3 transcription factor 3	0.17

		1834	CD33 antigen	0.17

	Wrapper	4847	**zxyin**	0.22

		3252	glutathione S-transferase	0.16

		6855	TCF3 transcription factor 3	0.11

Medulloblastoma	Greedy	5585	drebrin E	0.05

		4174	COL6A2 collagen type IV alpha 2	0.04

		3185	pancreatic beta cell growth factor	0.04

	Wrapper	2426	prostaglandin D2 synthase	0.04

		4710	acylphosphatase isozyme	0.03

		3185	pancreatic beta cell growth factor	0.03

Prostate cancer	Greedy	6185	**serine protease hepsin**	0.70

		8965	mitochondrial matrix protein P1	0.09

		10494	mRNA, ne1-related protein P1	0.07

	Wrapper	6185	**serine protease hepsin**	0.31

		8965	mitochondrial matrix protein P1	0.09

		4365	T-cell receptor Ti gamma chain	0.07

Leukemia 2	Greedy	8828	**HLA class II alpha chain-like**	0.59

		9101	MHC class II lymphocyte antigen	0.23

		2610	mRNA for oct-binding factor	0.13

	Wrapper	8828	**HLA class II alpha chain-like**	0.41

		9101	MHC class II lymphocyte antigen	0.20

		2610	mRNA for oct-binding factor	0.17

Genes listed **in bold **occur most frequently and are discussed in the text.

## Discussion

The reason for using AUC to measure classification performance in the training sample is that it can be computed quickly under a binormal assumption and is familiar to many researchers. The threshold increase in AUC to add a gene to the classification rule of 0.02 for the greedy algorithm and 0.01 for the wrapper algorithm represents a small improvement in performance relative to the range of AUC from 0.5 to 1.00. The specified threshold increase in AUC is smaller with the wrapper than with the greedy algorithm because splitting of the training sample into a training-training sample and training-test sample with the wrapper avoids overfitting, unlike the case with the greedy algorithm in which the entire training sample is used both gene selection and evaluation. Investigating various values for the threshold increase in AUC to determine an optimal threshold increase in AUC is not desirable in this setting because it would require the use of the test sample for both rule selection and evaluation, which could bias the results.

Some centroid-based classifications of microarray data shrink centroids to the mean of the centroids and select genes based on soft thresholding [18]. However this procedure is not desirable for our goals because it "makes the class centroids look more similar to each other" [19] and typically selects many more genes than with our approach. Some classification rules are based on the connectivity of each gene in a network [20]. However this approach is not desirable for our goal of identifying the few genes most directly predictive of class as some highly connected genes may be selected due to multiple associations with many moderately predictive genes and not because they are highly predictive themselves.

With Goal 1 of rule discovery and testing for purposes of prediction, one should consider baseline clinical variables as well as microarray data when formulating a classification rule. Binary variables can be coded as 0 or 1. Ordered variables created from continuous variables, such as age categories, can be assigned the midpoint of each category. An ordered variable of low, medium, and high can be treated as two binary variables, one comparing low versus medium and high, and one comparing low and medium versus high. To evaluate the use of classification rules to stratify patients for treatment, in a new sample one could select patients with the highest class probabilities based on the classification rule and randomize them to treatment.

With Goal 2 of gene discovery, the most frequently occurring genes (desmin, zyxin, hepsin, and HLA class II) among random splits of the training sample in the four data sets with good classification performance in the test samples have an interesting connection to the tissue organization field theory of carcinogenesis. Tissue organization field theory postulates that a disruption of intercellular communication between the microenvironment and the cells in which cancer arises is the proximal cause of cancer [21-23]. In contrast the somatic mutation theory postulates that genetic alterations in the cells in which cancer arises are the proximal cause of cancer. Desimin is associated with pericytes, cells in the blood vessel walls, that have been implicated in foreign-body carcinogenesis [24], a phenomenon that likely involves disruption of intercellular communication [25]. Zxyin is associated with morphogenesis [26]. Hepsin mediates the digestion of extracellular matrix components in initial tumor growth [27]. Lastly HLA class II is a marker for tumor-infiltrating dendritic cells [28]. These genes involve changes in the tumor microenvironment, which is important to the development of cancer under the tissue organization field theory.

## Conclusion

The proposed simple and flexible classification rule that select Swirls or Ripples after parsimoniously selecting genes and a distance measure is a good basis for either rule discovery and testing or gene discovery.

## Methods

### Computing the centroid set in the training sample

Let *z*_*TR*(*ijk*) _denote the gene expression level for gene *j *in specimen *i *of class *k *of the training sample Let *n*_*TR*(k) _denote the number of specimens in class *k *of the training sample. The centroid set *C *in the training sample is

(5) $$\begin{array}{l} C_{TR}=\left\{c_{TR(jk)},v_{TR(jk)},n_{TR(k)}\right\},\;\;\text{where}\\ c_{TR(jk)}=\sum_{i}\frac{z_{TR(ijk)}}{n_{TR(k)}},\;v_{TR(jk)}=\sum_{i}\frac{{\left(z_{TR(ijk)}-c_{TR(jk)}\right)}^{2}}{n_{TR(k)}-1}. \end{array}$$

### Measuring classification performance using AUC

Selection of the gene *G*, set distance measure *D*, and score formula *S *for the training sample involve the AUC under a binormal distribution [29] applied to the score,

(6) $$\begin{array}{l} AUCS(Z_{TR},F)\;=\;\Phi \left(\frac{m_{TR(0)}-m_{TR(1)}}{\sqrt{\Sigma_{k}w_{TR(k)}}}\right),\text{ where}\\ Z_{TR}=\text{\{}Z_{TR}{}_{\left(ik\right)}\text{\}, where }Z_{TR}{}_{\left(ik\right)}=\text{\{}z_{TR}{}_{\left(ijk\right)}\text{\}},\\ s_{TR}{}_{\left(ik\right)}=\text{Score (}Z_{TR}{}_{\left(ik\right)}\text{,}F),\\ m_{TR(k)}=\sum_{i}\frac{s_{(TR)ik}}{n_{k}}\;w_{TR(k)}=\sum_{i}\frac{{\left(s_{TR(ik)}-m_{TR(k)}\right)}^{2}}{n_{TR(k)}-1}, \end{array}$$

and Φ denotes the cumulative normal distribution function.

### Selecting the gene set, distance measure, score formula in the training sample

For each score formula *S *and each distance measure *D*, the classification rule selects a gene set *G*. To simplify notation, let *F*_*TR*{*a,b*} _= (*C*_*TR*_,*G *= {*a,b*}, *D, S*), where *a *and *b *refer to different genes. To make computations tractable, the classification rule starts with a preliminary filter that selects the 50 genes in the training sample with the highest values of *AUCS*(*Z*_*TR*_, *F*_*TR*{*j*}_), where *j *indexes genes. Subsequent calculations involve these 50 genes.

The greedy algorithm selects the gene *a *with the highest *AUCS*(*Z*_*TR*, _*F*_*TR*{*a*}_) and identifies the gene *b *with the highest *AUCS*(*Z*_*TR*_, *F*_*TR*{*a,b*}_). If the increase in AUC is less than 0.02, G = {*a*}; otherwise *G *includes {*a,b*} and this procedure continues for additional genes.

The wrapper algorithm involves five random splits, each with 50% of the training sample constituting a training-training sample (*TR*:*TR*) for formulating the classification rule and 50% constituting a training-test sample (*TR*:*TE*) for computing AUC. The algorithm selects the gene *a *with the highest *AUCS*(*Z*_*TR*:*TR*_, *F*_*TR*:*TE*{*a*}_) and identifies the gene *b *with the highest *AUCS*(*Z*_*TR*:*TR*_, *F*_*TR*:*TE*{*a,b*}_). If the increase in AUC is less than 0.01, *G *= {*a*}; otherwise *G *includes {*a,b*} and this procedure continues additional genes. The wrapper selects the best classification rule, in terms of AUC, among the random splits.

For each *S *with *G *already selected, the classification rule selects *D *= 1 if the increase in AUC for *D *= 2 is less than 0.02 and *D *= 2 otherwise. For each *S *with *D *and *G *already selected, the classification rule selects *S *with the highest AUC.

### Computing ROC and RU curves in the test sample

Let *F*_*TR *_denote the components of the final classification rule derived from the training sample. Let *z*_*TE*(*ijk*) _denote the gene expression level for gene *j *in specimen *i *of class *k *of the test sample, and let *Z*_*TE*(*ik*) _= {*z*_*TE*_(*ijk*)}. Let *n*_*TE*(*k*) _denote the number of specimens in class *k *of the test sample. At each cutpoint *u*, which corresponds to a decile of the combined distribution of gene expression levels over the two classes, the true positive rate (*TPR*) is the fraction of specimens from class 0 classified as 0, and the false positive rate (*FPR*) is the fraction of specimens from class 1 classified as 0,

(7) TPRu=∑idi0unTE(0), FPRu=∑idi1unTE(1), wherediku={1Score (ZTE(ik),FTR)<u,0otherwise.

For Goal 1, confidence intervals are computed by bootstrapping the data in the test sample 20 times. For each bootstrap sample, *TPR *at *FPR *= 0.1, 0.2, ..., 0.9 is computed via linear interpolation. The ROC curve plot for the bootstrap iterations consists of the mean ROC curve and upper and lower bounds based on the standard deviation of the ROC curves. An RU curve is computed from the concave envelope of the mean ROC curve, where the risk thresholds are derived from the slopes of the ROC curve. If the concave ROC curve has only one point between (0,0) and (1,1), there are insufficient data to compute a RU curve.

## Availability and requirements

Project name: Swirl

Project homepage:

http://prevention.cancer.gov/programs-resources/groups/b/software/swirls

Programming language: Mathematica 7.0 [30]

Disclaimer: This code is provided "as is", without warranty of any kind, express or implied, including but not limited to the warranties of merchantability, fitness for a particular purpose and noninfringement. In no event shall the National Cancer Institute or the individual developers be liable for any claim, damages or other liability of any kind. Use of this code by recipient is at recipient's own risk. The National Cancer Institute makes no representations that the use of the code will not infringe any patent or proprietary rights of third parties.

Reproducibility. Functions are provided to reproduce calculations.

Inputs: The user needs to specify (*a*) the gene expression data, consisting of two matrices, one for each class, with rows corresponding to genes and columns corresponding to specimens, (*b*) a list of gene names, the name of the data set, and names of the two classes, and (*c*) the class probabilities in the target population, if different from study population, for computing the RU curve.
